# In the presence of population structure: From genomics to candidate genes underlying local adaptation

**DOI:** 10.1002/ece3.6002

**Published:** 2020-02-12

**Authors:** Nicholas Price, Lua Lopez, Adrian E. Platts, Jesse R. Lasky

**Affiliations:** ^1^ Department of Bioagricultural Sciences & Pest Management Colorado State University Fort Collins CO USA; ^2^ Department of Biological Sciences University of Cyprus Nicosia Cyprus; ^3^ Department of Biology Binghamton University (State University of New York) Binghamton NY USA; ^4^ Simons Center for Quantitative Biology Cold Spring Harbor Laboratory Cold Spring Harbor NY USA; ^5^ Department of Biology Center for Genomics and Systems Biology New York University New York NY USA; ^6^ Department of Biology Pennsylvania State University University Park PA USA

**Keywords:** flowering time, population genomics, population structure, quantitative trait loci, selective constraint

## Abstract

Understanding the genomic signatures, genes, and traits underlying local adaptation of organisms to heterogeneous environments is of central importance to the field evolutionary biology. To identify loci underlying local adaptation, models that combine allelic and environmental variation while controlling for the effects of population structure have emerged as the method of choice. Despite being evaluated in simulation studies, there has not been a thorough investigation of empirical evidence supporting local adaptation across these alleles. To evaluate these methods, we use 875 *Arabidopsis thaliana* Eurasian accessions and two mixed models (GEMMA and LFMM) to identify candidate SNPs underlying local adaptation to climate. Subsequently, to assess evidence of local adaptation and function among significant SNPs, we examine allele frequency differentiation and recent selection across Eurasian populations, in addition to their distribution along quantitative trait loci (QTL) explaining fitness variation between Italy and Sweden populations and cis‐regulatory/nonsynonymous sites showing significant selective constraint. Our results indicate that significant LFMM/GEMMA SNPs show low allele frequency differentiation and linkage disequilibrium across locally adapted Italy and Sweden populations, in addition to a poor association with fitness QTL peaks (highest logarithm of odds score). Furthermore, when examining derived allele frequencies across the Eurasian range, we find that these SNPs are enriched in low‐frequency variants that show very large climatic differentiation but low levels of linkage disequilibrium. These results suggest that their enrichment along putative functional sites most likely represents deleterious variation that is independent of local adaptation. Among all the genomic signatures examined, only SNPs showing high absolute allele frequency differentiation (AFD) and linkage disequilibrium (LD) between Italy and Sweden populations showed a strong association with fitness QTL peaks and were enriched along selectively constrained cis‐regulatory/nonsynonymous sites. Using these SNPs, we find strong evidence linking flowering time, freezing tolerance, and the abscisic‐acid pathway to local adaptation.

## Graphical abstract

Alleles underlying local adaptation are expected to: (a) be enriched along regions explaining fitness variation between populations; (b) exhibit population genomic signatures of local adaptation and selection; and (c) show evidence of function. In Arabidopsis thaliana, when examining populations that are isolated by large geographical distances and inhabiting different climates, genotype by environment association methods perform poorly in satisfying the above assumptions. Among the methods examined, SNPs showing high allele frequency divergence and linkage disequilibrium satisfied all key points (a, b, and c). Using these SNPs, we find strong evidence linking flowering time, and other interesting life‐history traits to climate adaptation.

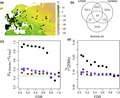

## 1 INTRODUCTION

Populations of a species may inhabit different environments where local selection pressures favor a combination of (multivariate) phenotypes (Conover, Duffy, & Hice, 2009; Hereford, 2009; Leimu & Fischer, 2008; Savolainen, Lascoux, & Merila, 2013). Local adaptation, by definition, occurs when the resident genotype is expected, on average, to have a higher relative fitness than a foreign genotype (Kawecki & Ebert, 2004). Despite the widespread evidence of local adaptation in many taxa (Arguello et al., 2016; Jeong & Di Rienzo, 2014; Leimu & Fischer, 2008), our understanding of the traits involved, its genetic basis, and its environmental underpinnings is still at an infant stage (Wadgymar et al., 2017, Tiffin & Ross‐Ibarra, 2014, Savolainen et al., 2013).

In a variety of species, reciprocal transplant and common garden/laboratory experiments have showed significant adaptive differentiation between natural populations inhabiting different environments (Ågren & Schemske, 2012; Hendry, Taylor, & Mcphail, 2002; Kaufmann, Lenz, Kalbe, Milinski, & Eizaguirre, 2017; Phifer‐Rixey et al., 2018; Savolainen, Pyhäjärvi, & Knürr, 2007; Via, 1991). Furthermore, in plants and animals, mapping experiments have uncovered quantitative trait loci (QTL) for traits that are thought to underlie local adaptation (Ågren, Oakley, Lundemo, & Schemske, 2017; Colosimo et al., 2004; Oakley, Agren, Atchison, & Schemske, 2014; Yang, Guo, Shikano, Liu, & Merila, 2016), in addition to QTL explaining fitness differences across environments (Ågren, Oakley, McKay, Lovell, & Schemske, 2013; Anderson, Lee, Rushworth, Colautti, & Mitchell‐Olds, 2013). Despite the importance of QTL studies in providing direct evidence for local adaptation (Ågren et al., 2013), in many instances they provide a low resolution for its genetic basis, and in practical terms are time‐consuming, expensive, and labor‐intense (Joosen, Ligterink, Hilhorst, & Keurentjes, 2009).

With the advent of low‐cost, and fast, next‐generation sequencing (Henson, Tischler, & Ning, 2012), higher‐resolution population genomic approaches have emerged as the new means for examining the genetic basis of local adaptation (Tiffin & Ross‐Ibarra, 2014), (Lachance & Tishkoff, 2013; Savolainen et al., 2013; Sork, 2017). In brief, these methods include (a) identifying single nucleotide polymorphisms (SNPs) showing significant allele frequency differentiation between populations (*F*_ST_) (Beaumont & Balding, 2004; De Villemereuil & Gaggiotti, 2015; Foll & Gaggiotti, 2008); (b) identifying genomic regions showing significant increases in linkage disequilibrium (Jacobs, Sluckin, & Kivisild, 2016) or composite likelihood ratios for recent sweeps (Degiorgio, Huber, Hubisz, Hellmann, & Nielsen, 2016; Huber, Degiorgio, Hellmann, & Nielsen, 2016); and (c) identifying genotype‐by‐environment associations (GEA; Caye, Jumentier, Lepeule, & Francois, 2019; Gunther & Coop, 2013; Lasky et al., 2012; Luu, Bazin, & Blum, 2017; Zhou & Stephens, 2012). The latter approach has gained attention because it can be implemented based on individual (as opposed to population‐based) sampling, and furthermore, it provides a direct link to ecologically relevant factors (e.g., climate).

An important hurdle that GEA and other methods need to overcome, is disentangling the effects of selection from demographic history (Hoban et al., 2016; Lotterhos & Whitlock, 2014). While nonadaptive processes (Bierne, Welch, Loire, Bonhomme, & David, 2011; Bonhomme et al., 2010; Hofer, Ray, Wegmann, & Excoffier, 2009) can generate population structure along the genome, and therefore lead to spurious genotype‐by‐environment associations, geographically varying environments can generate population structure in the regions of genes involved in adaptation (Mckay and Latta, (2002). To limit the number of spurious associations, studies usually estimate population structure using different methods (Price, Zaitlen, Reich, & Patterson, 2010), incorporate population structure (Kang et al., 2010; Wang et al., 2011; Yu et al., 2006; Zhou & Stephens, 2012) and/or geographic structure (Lasky et al., 2012) into statistical models, and finally test whether certain loci explain significantly higher variation in environment/climate than population structure itself (Fischer et al., 2013; Frachon et al., 2018; Hancock, Brachi, et al., 2011; Huber, Nordborg, Hermisson, & Hellmann, 2014; Lasky et al., 2014, 2012; Lasky, Forester, & Reimherr, 2018; Monroe et al., 2016; Price et al., 2018; Rellstab et al., 2017). While such approaches may limit the number of false positives, they can also lead to false negatives (Anderson, Willis, & Mitchell‐Olds, 2011; Bergelson & Roux, 2010) since adaptive variants can also generate population structure, or can be randomly correlated with population structure.

To examine the performance of population genomic methods (primarily *F*_ST_‐based and GEA methods), there have been a multitude of simulation studies examining different demographic scenarios and selection regimes (De Mita et al., 2013; De Villemereuil, Frichot, Bazin, François, & Gaggiotti, 2014; Forester, Lasky, Wagner, & Urban, 2018; Lotterhos & Whitlock, 2014, 2015; Perez‐Figueroa, Garcia‐Pereira, Saura, Rolan‐Alvarez, & Caballero, 2010; Yoder & Tiffin, 2017). Some of the general findings of these studies are (a) that the underlying demographic history can have a significant impact on which approach (*F*_ST_ or GEA) performs better (Lotterhos & Whitlock, 2015); (b) that under certain demographic scenarios the power of all methods can be low (De Mita et al., 2013; De Villemereuil et al., 2014); (c) the intensity/timing of selection, in addition to the number of loci involved, can have a significant impact on performance (De Villemereuil et al., 2014; Forester et al., 2018; Yoder & Tiffin, 2017); and (d) the sampling of individuals and markers can have a significant impact on the power to detect causative loci (De Mita et al., 2013; Yoder & Tiffin, 2017).

While simulation studies provide a controlled environment to evaluate methodologies, and determine the factors that can affect each method, we do not know how well they emulate real data and the complex demographic and selection forces underlying them. To compensate for that, the current study compares various empirical evidence of local adaptation, recent selection, and function, to provide a general evaluation for each approach. As a study system, we use the species *Arabidopsis thaliana,* hereafter mentioned as Arabidopsis. Arabidopsis populations are found all across Eurasia (1001 Genomes Consortium, 2016), traversing very different climates (Frachon et al., 2018; Lasky et al., 2012; Mojica et al., 2016; Monroe et al., 2018), and therefore have been thought to be locally adapted. Direct evidence of local adaptation has been observed between North Sweden and Central Italy populations (Ågren & Schemske, 2012; Fournier‐Level et al., 2011), in which the fitness of genotypes was measured in a reciprocal transplant experiment at the native sites of each population (Ågren & Schemske, 2012); and recombinant inbred lines of these were used to map quantitative trait loci (QTL) explaining fitness variation (Ågren et al., 2013). Such QTL, can be used to evaluate population genomic methods by testing whether candidate SNPs are enriched within their confidence intervals. Furthemore, given that most of these QTL are of low resolution and span large genomic regions, a tight association between candidate SNPs and logarithm of the odds ratio (LOD) peaks should provide stronger support for an approach. In addition to a tight association to fitness QTL, when the underlying loci exhibit fitness trade‐offs (i.e., when one allele is advantageous in one environment but deleterious in another) we would expect significant allele frequency differentiation between locally adapted populations (Tiffin & Ross‐Ibarra, 2014). Allele frequency differentiation is also expected under conditional neutrality (i.e., when one allele is advantageous in one environment but neutral in another) but to a lesser degree (Tiffin & Ross‐Ibarra, 2014). GEAs are examined using a large sample of individuals across multiple populations and environments to identify candidate SNPs underlying local adaptation. If a large proportion of these SNPs includes causal or linked loci, then we would expect them to show significant allele frequency divergence between pairs of locally adapted populations and significant enrichment along fitness QTL peaks. Furthermore, if selection is relatively recent, candidate SNPs of both *F*_ST_‐ and GEA‐based methods are expected to be within genomic regions that exhibit high linkage disequilibrium (Charlesworth, 2006; Kim & Nielsen, 2004) and a site frequency spectrum that is expected under a recent selective sweep (Kim & Stephan, 2002; Nielsen et al., 2005).

In conjunction with population genomic signatures of selection or significant associations with climate, genetic variation underlying local adaptation is expected to be enriched along sites that are functional and influence fitness. SNPs showing significant associations with climate were found to be significantly enriched among nonsynonymous, but also synonymous variation (Hancock, Brachi, et al., 2011; Lasky et al., 2012). The enrichment among synonymous variation (which largely evolve neutrally) may be the result of linkage disequilibrium due to neutral processes but also background selection (Charlesworth, Morgan, & Charlesworth, 1993) and/or hitchhiking (Gillespie, 2000). A stricter enrichment test will be one that controls for sequence conservation along coding and noncoding sites. Sites that are highly conserved among species (Haudry et al., 2013; Hupalo & Kern, 2013; Miller et al., 2007) are assumed to be under functional constraint and selectively important—that is, due to purifying selection the number of tolerated mutations is limited (Graur, 2016). Therefore, SNPs showing significant evidence of local adaptation across highly constraint sites are more likely to be true positives.

To provide an evaluation of the approaches frequently used to study the genetic basis of local adaptation, we include two methods that identify genotype‐by‐environment associations while accounting for population structure [genome‐wide efficient mixed model (GEMMA; Zhou & Stephens, 2012) and the latent factor mixed model (LFMM; Caye et al., 2019)] and a method [BAYESCAN (Foll & Gaggiotti, 2008)] that identifies SNPs showing higher allele frequency differentiation than expected under various neutral models of evolution. The two GEA methods (GEMMA and LFMM) were applied across a set of 875 Eurasian accessions (1001 Genomes Consortium, 2016) and four climate variables covering temperature, precipitation and photosynthetically active radiation (Lasky et al., 2012; Price et al., 2018). The 875 accessions excluded likely laboratory escapees or contaminants (Pisupati et al., 2017) and invasive lines that may reduce the signal of local adaptation (Lasky et al., 2012). BAYESCAN on the other hand was applied to a sample of accessions in North Sweden and South Italy.

After obtaining the results, we addressed the following questions: What is the overlap in significant SNPs identified by all methods? Do they show significant allele frequency differentiation and linkage disequilibrium between locally adapted Sweden and Italy populations? What are the derived allele frequency spectra and levels of linkage of disequilibrium of these SNPs across the whole Eurasian range? Do SNPs identified by any of the methods show a significant association/enrichment with and along fitness QTL peaks? Finally, do any of these SNPs show enrichment along cis‐regulatory/nonsynonymous sites showing significant functional constraint?

Addressing the above questions allowed us to assess these methods and consider the best approach to examine candidate genetic variation underlying 20 fitness QTL and evidence tying flowering time to local adaptation. Flowering time is a life‐history trait that is thought to play a significant role in local adaptation to climate (Ågren et al., 2017); Dittmar, Oakley, Agren, & Schemske, 2014; Hall & Willis, 2006; Sandring & Agren, 2009; Verhoeven, Poorter, Nevo, & Biere, 2008), and whose genetic basis has been thoroughly studied (Salomé et al., 2011; Sasaki et al., 2015). To re‐examine evidence linking flowering time to climate adaptation, we used the following data: (a) a list of genes that were experimentally shown to affect flowering time; (b) high‐confidence QTL explaining flowering time variation between Italy and Sweden populations (Ågren et al., 2017), and (c) flowering time estimates for Arabidopsis Eurasian accessions (1001 Genomes Consortium, 2016).

## 2 MATERIALS AND METHODS

### 2.1 Identifying SNPs showing significant associations with climate after accounting for population structure

To identify SNPs showing significant associations with climate, while accounting for population structure we used two prominent methods: GEMMA association (Zhou & Stephens, 2012) and LFMM (Caye et al., 2019), version 2. These methods were applied to four climate variables [Minimum Temperature of Coldest Month; Precipitation during Warmest Quarter; Soil moisture; and Photosynthetically Active Radiation during Fall (which is the time of germination for fall ecotypes such as in Italy and Sweden)] important to local adaptation (Lasky et al., 2012), and a SNP genotype matrix (1001 Genomes Consortium, 2016) derived from a set of 875 re‐sequenced *Arabidopsis thaliana* Eurasian accessions (Table S1) that excluded laboratory escapees/contaminants (Pisupati et al., 2017) and accessions from outside the native Eurasian and African range of *A. thaliana* that may have weaker patterns of local adaptation (Lasky et al., 2012).

After we filtered for biallelic SNPs with minor allele frequency >0.05, we tested association with home climate of ecotype and tested for potential confounding effects of population structure using the software GEMMA (Zhou & Stephens, 2012) with a missingness threshold of 0.05. For the linear mixed model option, we used the Wald test (default) to test the null hypothesis that the mean climate occupied by the two alleles is equal (Lasky et al., 2014). To apply LFMM, we first used the R function “prcomp,” to perform a principal component analysis (PCA) and estimate structure in the genotypic data. “prcomp” was applied over 100 random samples of 20,000 polymorphic loci. After determining how many components (K) explained most of the genotypic variance, we applied LFMM (“lfmm_test”) in R and calibrated p‐values using the “gif” option. To estimate a false discovery rate for both GEMMA and LFMM, we used the “qvalue” function (Storey, 2002) implemented in R.

### 2.2 Estimating allele frequency differentiation and recent selection across Arabidopsis thaliana populations

Allele frequency differentiation between 25 South Italy and 40 North Sweden accessions (Tables S2 and S3) was estimated using an *F*_ST_‐based metric implemented in BAYESCAN (Foll & Gaggiotti, 2008) and a simple measure of absolute allele frequency differentiation (|*f*_N.Sweden_ – *f*_S.Italy_|). |*f*_N.Sweden_ – *f*_S.Italy_| was used as an alternative measure, because from prior experience (Price et al., 2018) we noticed that significant *F*_ST_s were limited to SNPs in which alternative alleles were fixed between populations (>0.95).

In addition to allele frequency differentiation between Italy and Sweden populations, we also estimated derived allele frequencies across the set of 875 Eurasian accessions (DAF_eurasia_) for SNPs that we could estimate the ancestral state. To infer an ancestral base probability, we used Phast suite's Prequel function (Hubisz, Pollard, & Siepel, 2011), and a MAF genome alignment of the species *Arabidopsis thaliana* (AT) (Berardini et al., 2015), *Arabidopsis lyrata* (AL) (Hu et al., 2011), *Arabidopsis haleri* (AH) (Briskine et al., 2017), *Capsella rubella* (CR) (Slotte et al., 2013), and *Neslia paniculata* (NP). Alignments were generated with LASTZ (Harris, 2007) and refined for high‐confidence orthologs using the pipeline described by (Haudry et al., 2013). In order to remove reference bias, the *A. thaliana* genome was neutralized in the alignments before calling the ancestral base. The neutral tree used for ancestral inference was ((AT:0.0640717,(AH:0.0239032,AL:0.0287045):0.029485):0.0306753,(NP:0.0654492,CR:0.0837745):0.0306753)ANC, where ANC is the location of the simulated ancestor. Derived allele frequencies were estimated when an ancestral state had a probability >0.6.

To estimate evidence of recent selection at the SNP level, we computed linkage disequilibrium (LD) using the coefficient of correlation (*r* ^2^). More specifically, using the package “PLINK” (Purcell et al., 2007) LD at a given SNP was estimated as the mean *r* ^2^ between it and neighboring SNPs within a 20‐kb window ($\bar{r^{2}}$). LD was measured using the 65 Italy–Sweden accessions and the 875 Eurasian accessions ($\bar{r_{\text{eurasia}}^{2}}$). As an additional measure of local adaptation across Italy and Sweden populations, we used SNPs that showed a high |*f*_N.Sweden_ – *f*_S.Italy_| > 0.70 and LD > 0.19 (hereafter referred to as AFD.LD SNPs). 0.70 and 0.19 represent the 95th percentiles of the respective genome‐wide distributions.

For evidence of recent sweeps, we used previously calculated (Price et al., 2018) composite likelihood ratios (CLRs) that were computed using Sweepfinder2 (Degiorgio et al., 2016). We focused on CLRs in North Sweden, since preciously estimated CLR signals in other populations were very weak (Huber et al., 2014; Long et al., 2013; Price et al., 2018).

### 2.3 Determining sites of functional importance

To narrow down SNPs to ones that are more likely to underlie differences in function/expression of protein‐coding genes, we focused on cis‐regulatory and nonsynonymous variation that was found along sites showing significant selective constraint. We regarded cis‐regulatory SNPs as those found within 1 kb upstream from the transcriptional start site of a gene (Pass et al., 2017; Zou et al., 2011), unless these sites were found in genic regions of other genes (in which case they were excluded). To call nonsynonymous variation, we used biallelic sites, we used a publicly available python script (callSynNonSyn.py; archived at https://github.com/kern-lab/), and gene models downloaded from the TAIR database (TAR10 genome release) (Berardini et al., 2015). To annotate regions showing significant selective constraint across the *A. thaliana* genome, we used phastCons scores (Siepel et al., 2005) derived using a nine‐way alignment of Brassicaceae species from the study by (Haudry et al., 2013). We defined conserved regions as those with a score ≥0.8 over blocks of ≥10 nucleotides.

### 2.4 Fitness and flowering time QTL underlying Italy and Sweden populations

Quantitative trait loci explaining fitness variation between natural *A. thaliana* Italy and Sweden populations were retrieved by the study of (Ågren et al., 2013). These 20 fitness QTL (Table S4) were assembled into 6 genetic trade‐off QTL (Ågren et al., 2013); however, we treated them as independent given the very long genetic distances between fitness QTL peaks (Table S4). Furthermore, high‐confidence QTL explaining flowering time variation were retrieved between these populations were retried from (Ågren et al., 2017) (Table S5).

### 2.5 Estimating enrichment of candidate SNPs along functional sites and fitness QTL

To test whether SNPs showing significant evidence of local adaptation are enriched along fitness QTL peaks and among cis‐regulatory/nonsynonymous variation at sites showing significant selective constraint, we used 1,000 circular permutations. For example, when examining whether SNPs showing significant correlations to climate are enriched within a given distance from fitness QTL peaks or cis‐regulatory/nonsynonymous sites we execute the following steps:

1. Shifting the *q*‐values along the genome while keeping the genomic positions intact. During this step, we take the whole list of *q*‐values (“qvalues”) and shift them circularly from a random location (“random_loc”) using the following R‐code: shifted_qvalues < −qvalues[c(random_loc:total_no_SNPs, 1:( random_loc − 1))]. This shifts the tail‐end *q*‐values to the beginning of the list and vice versa.
2. We then choose significant SNPs using our threshold for significance (FDR/*q*‐value <0.1) and estimate the proportion of SNPs within a given distance from a fitness QTL peak (e.g., 100 kb) or the proportion of SNPs at cis‐regulatory/nonsynonymous sites showing significant functional constraint.
3. The above two steps are repeated 1,000 times to build a distribution of expected proportions. These distributions are compared to the observed proportion.

The same approximate procedure was followed when estimating the expected proportions of SNPs showing a significant *F_ST_* according to BAYESCAN or SNPs showing a high absolute allele frequency divergence and linkage disequilibrium across Italy and Sweden populations (AFD.LD).

### 2.6 Sliding window analysis of chromosomal variation SNPs showing evidence of local adaptation

To detect chromosomal regions with a high proportion of SNPs showing significant evidence of local adaptation, we used a sliding window approach. Specifically, for a window size of 20kb and a step size of 1kb we estimated the ratio of SNPs showing a specific requirement (e.g., |*f*_N.Sweden_ – *f*_S.Italy_| > 0.70 and LD > 0.19) over the total number of SNPs within a 20‐kb window.

### 2.7 Flowering time estimates for A. thaliana Eurasian accessions and candidate flowering time genes

Estimates of flowering time for the 835 Eurasian *A. thaliana* accessions were downloaded from the study by the 1001 Genomes Consortium (1001 Genomes Consortium, 2016). In brief, plants were grown in growth chambers with the following settings: after 6 days of stratification in the dark at 4°C, constant temperature of 16°C with 16 hr of light/8 hr of darkness, and 65% humidity. Flowering time was scored as days until first open flower. See 1001 Genomes Consortium (2016) for further details. A set of genes that were experimentally verified to affect flowering time was downloaded from Prof. Dr. George Coupland website (Table S6). (https://www.mpipz.mpg.de/14637/Arabidopsis_flowering_genes).

### 2.8 Constructing rooted gene trees

To build neighbor‐joining trees of genes showing significant local adaptation, we downloaded 1:1 orthologs between *Arabidopsis thaliana* and outgroups *Arabidopsis lyrata* and *Capsella rubella* from the Phytozome database (Goodstein et al., 2012) and after aligning the coding sequences with MAFFT (Katoh & Toh, 2008) we used MEGA (Tamura, Stecher, Peterson, Filipski, & Kumar, 2013) to build rooted gene trees.

## 3 RESULTS

### 3.1 Climate‐associated SNPs show low allele frequency differentiation and linkage disequilibrium across Italy and Sweden populations

To identify SNPs showing significant associations with climate while accounting for population structure, we applied GEMMA (Zhou & Stephens, 2012) and LFMM 2 (Caye et al., 2019) on 875 Eurasian accessions (Table S1) and four climate variables (Minimum Temperature of Coldest Month; Annual mean temperature; Precipitation during warmest quarter; and Photosynthetically active radiation during fall [time of germination of Italy and Sweden ecotypes (Ågren & Schemske, 2012)]. In addition to identifying SNPs showing significant associations with climate, we also applied BAYESCAN (Foll & Gaggiotti, 2008) between 40 accessions North Sweden and 25 accessions in South Italy (Tables S2 and S3) (referred to as Sweden and Italy hereafter) to identify SNPs that showed significant allele frequency differentiation estimated by *F*_ST_ after accounting for different models of neutral evolution.

**Figure 1:**
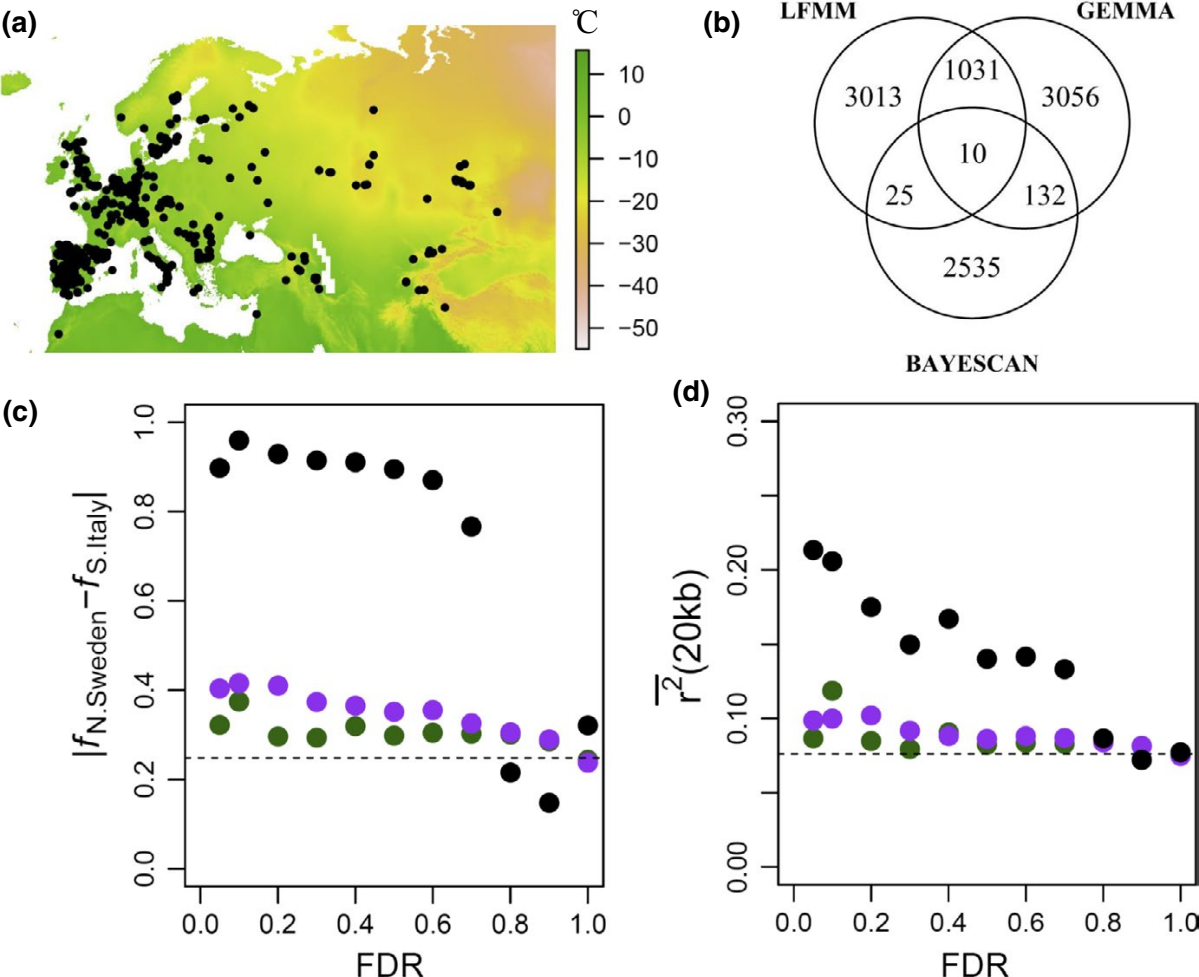
Low genetic divergence and recent selection underlying climate‐associated SNPs in locally adapted Italy and Sweden populations (Ågren & Schemske, 2012; Ågren et al., 2013). (a) Map depicting Minimum Temperature of Coldest Month (Min.Tmp.Cld.M) across the native range of 875 re‐sequenced *Arabidopsis thaliana* accessions (black dots). (b) A Venn diagram depicting the overlap in SNPs that showed significant associations with Min.Tmp.Cld.M according to LFMM and GEMMA, in addition to SNPs that showed significant allele frequency differentiation (*F*_ST_) according to BAYESCAN. Significance was set at an FDR < 0.1, and climate‐associated SNPs were filtered for ones that segregated between Italy and Sweden populations. (c) Examining the trend between absolute allele frequency divergence (AFD: |*f*_N.Sweden_ – *f*_S.Italy_|) and false discovery rate (FDR) when using GEMMA (green dots); LFMM (purple dots); and BAYESCAN (black dots). The first two points are separated by an interval of 0.05 and the rest by an interval of 0.1. The dotted line depicts the genome average. (d) Similarly, to Figure 1c, this figure examines linkage disequilibrium (LD) across Italy and Sweden populations. LD was estimated at the SNP level by taking the average *r* ^2^ between a SNP and all other SNPs within a 20‐kb window [*r* ^2^ (20 kb)]

After examining the results. we focused on Minimum Temperature of Coldest Month (Min.Tmp.Cld.M) (Figure 1a) because the other climate variables resulted in a very small number of significant SNPs [similar to a previous study (Price et al., 2018)] and in many instances did not a show an enrichment of low *p*‐values when examining their *p*‐value histograms. Furthermore, winter temperatures, which are approximated by Min.Tmp.Cld.M, have been shown to play an important role in local adaptation of Arabidopsis (Ågren & Schemske, 2012; Gienapp et al., 2017; Lasky et al., 2018; Oakley et al., 2014).

As depicted in appendices 1 and 2, the p‐value histograms produced by GEMMA and LFMM showed an enrichment of low p‐values and relatively uniform distributions at larger values. LFMM was applied using eight latent factors (*K* = 8) to account for population structure (Appendix S2). Using a *K* = 8 showed the most uniformity across large p‐values (Appendix S2) and resulted in a similar number of significant SNPs with GEMMA (Figure 1b). Smaller *K*s resulted in a disproportionally larger number of significant SNPs and not a very uniform distribution across large *p*‐values.

When examining significant SNPs (FDR < 0.1) after filtering out any significant associations with climate that did not segregate between Italy and Sweden populations, we find a very small overlap between methodologies (Figure 1b). The two GEA methods used (GEMMA and LFMM) showed a 25% overlap in predicted SNPs (Figure 1b), in addition to a negligible overlap with SNPs that showed significant allele frequency differentiation (*F*_ST_) between Italy and Sweden populations according to BAYESCAN (Figure 1b). It is quite surprising that LFMM and GEMMA captured an extremely small proportion of SNPs that showed a significant *F*_ST_ between Sweden and Italy populations (Figure 1b) given the strong adaptive divergence and evidence of genetic trade‐offs underlying these populations (Ågren et al., 2013; Ågren & Schemske, 2012). This, however, may occur because BAYESCAN chooses a very different set of SNPs with a high *F*_ST_.

To further examine the evidence of recent selection and genetic differentiation captured by the three methods in Italy and Sweden, we looked at absolute allele frequency differentiation (AFD: |*f*_N.Sweden_ – *f*_S.Italy_|) and linkage disequilibrium (LD) estimated as the SNP level ($\bar{r^{2}}$ (20 kb)—the average *r* ^2^ between a SNP and its neighboring SNPs within 20 kb). These measures were examined across different false discovery rates (FDRs), in order to study the relation between these signatures of adaptation and FDR (Figure 1c,d).

As expected, SNPs showing a significant *F*_ST_ (FDR < 0.1) according to BAYESCAN also showed a very high |*f*_N.Sweden_ – *f*_S.Italy_| (Figure 1c). A high |*f*_N.Sweden_ – *f*_S.Italy_| was also observed across SNPs that showed a higher FDR (Figure 1c), but that significantly dropped at an FDR > 0.7, where 98.5% of the SNPs were found. On the contrary, |*f*_N.Sweden_ – *f*_S.Italy_| associated with GEMMA and LFMM SNPs was low and near the genomic average across all FDRs (Figure 1c). The same pattern was observed when examining LD, where climate‐associated SNPs showed a very low LD that was repeated across all FDRs (Figure 1d), while SNPs showing a significant *F*_ST_ according to BAYESCAN showed a significantly higher LD than the genome average (Figure 1d), and as expected, LD decreased as FDR increased. All in all, these results indicate that climate‐associated SNPs capture very little evidence of local adaptation and recent selection across Italy and Sweden populations.

### 3.2 Derived allele frequencies, linkage disequilibrium, and climatic differentiation, across Eurasian populations, significantly differ among candidate SNPs

The weak evidence of local adaptation observed across climate‐associated SNPs may occur because they capture selection in other parts of the species range. To examine this possibility, we looked at their derived allele frequencies across the Eurasian range (DAF_eurasia_), in association with linkage disequilibrium ($\bar{r_{\text{eurasia}}^{2}}$ (20 kb). Furthermore, we compared DAF_eurasia_ and $\bar{r_{\text{eurasia}}^{2}}$ between climate‐associated SNPs and SNPs that showed significant allele frequency differentiation and LD between Italy and Sweden populations. More specifically, the SNPs included were (a) all significant (FDR < 0.1) LFMM and GEMMA SNPs (i.e., unlike Figure 1b, we included SNPs that did not segregate between Italy and Sweden populations; (b) SNPs that exhibited significantly high *F*_ST_ *s* between Italy and Sweden populations according to BAYESCAN; and (c) SNPs that showed a high AFD and LD between Italy and Sweden populations (hereafter referred to as “*AFD*.LD”). This latter measure was included because 92% BAYESCAN SNPs showed an |*f*_N.Sweden_ – *f*_S.Italy_| ≈ 1, and we wanted to include SNPs that showed lower divergences and included high levels of linkage disequilibrium [comparisons of a similar measure of AFD and *F*_ST_ are discussed in Ref. (Berner, 2019)]. As a threshold for high, we used the 95th percentiles of the |*f*_N.Sweden_ – *f*_S.Italy_| and LD distributions (|*f*_N.Sweden_ – *f*_S.Italy_| > 0.70 & LD > 0.19).

**Figure 2:**
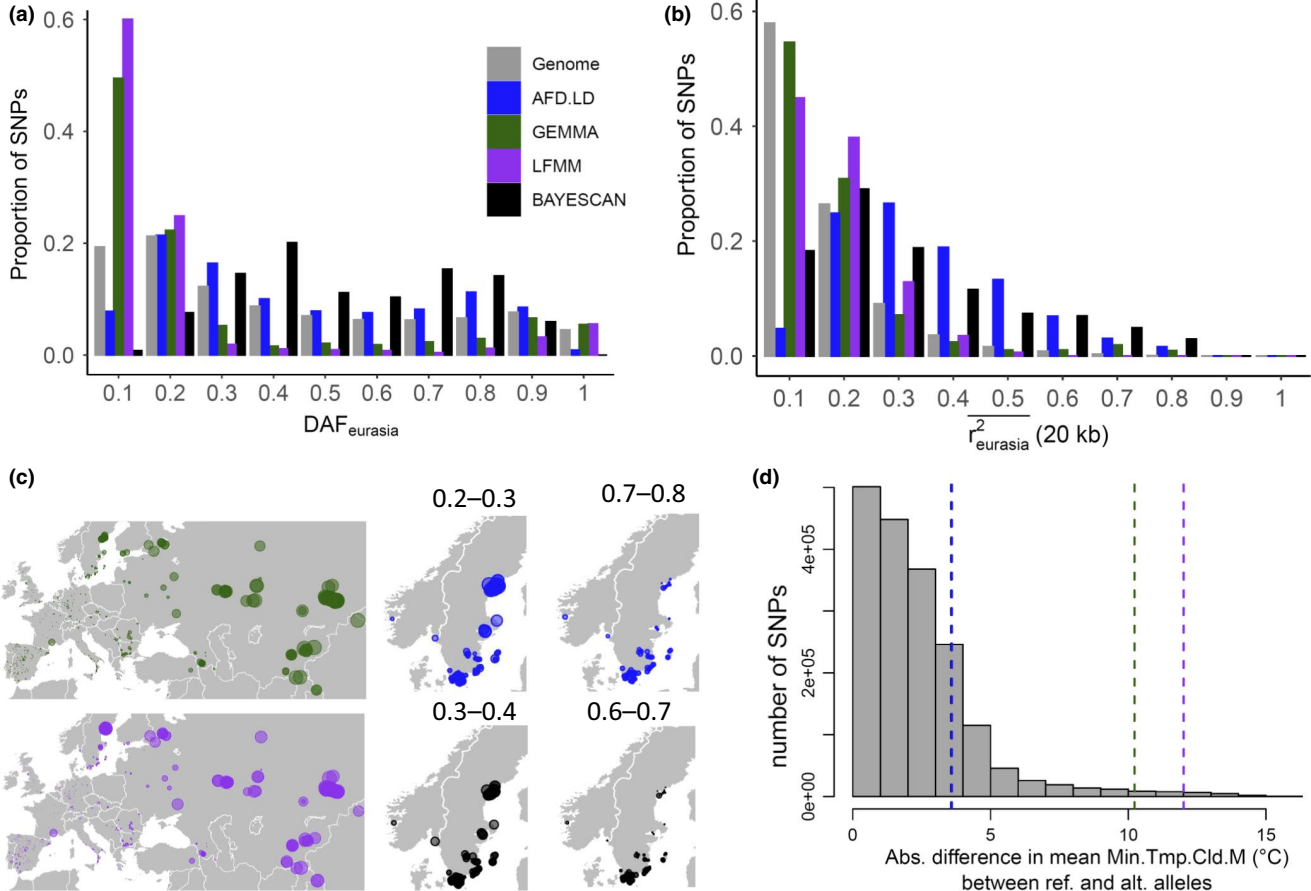
Candidate SNPs associated with local adaptation show contrasting patterns in genetic differentiation, selection, and climatic properties across Eurasia. (a) Comparing the derived allele frequency spectra (DAF_eurasia_) of candidate SNPs underlying local adaptation (GEMMA, LFMM, BAYESCAN, AFD.LD) and a genome‐wide set (Genome). (b) Comparing linkage disequilibrium [$\bar{r_{\text{eurasia}}^{2}}$ (20 kb)] across the different classes of SNPs. (c) Examining the location of candidate SNPs with DAFs that showed the highest enrichment relative to the genome‐wide set of SNPs (Figure 2a): AFD.LD (02–0.3 and 0.7–0.8); BAYESCAN (0.3–0.4 and 0.6–0.7); LFMM (<0.1); and GEMMA (<0.1). The size of the circles corresponds to the frequency of derived alleles at a specific location. (d) Absolute difference in mean Min.Tmp.Cld.M between reference and alternative alleles of candidate SNPs. AFD.LD and BAYESCAN SNPs show a smaller difference in Min.Tmp.Cld.*M* (~3.6°C) than GEMMA and LFMM SNPs (~10°C)

After estimating the ancestral nucleotide states across the *A. thaliana* genome (explained in Materials and Methods), we estimated DAF_eurasia_ for a subset of SNPs: LFMM (2,438), GEMMA (1,899), BAYESCAN (502), and *AFD.LD* (9,941). As shown in Figure 2a, compared to the DAF across the genome (“Genome”), GEMMA and LFMM are significantly enriched at low‐derived frequency variants (0.05 ≤ DAF_eurasia_ <0.1—SNPs with a MAF < 0.05 were filtered out before examination). Contrary to LFMM and GEMMA, BAYESCAN and *AFD.LD* SNPs showed a significant depletion in low‐frequency variants and a higher proportion DAFs between 0.2–0.4 and 0.6–0.8 (Figure 2a). These results indicate that SNPs showing significant allele differentiation and LD between Italy and Sweden populations show higher DAFs across the whole Eurasian range, when compared to SNPs showing significant associations with climate.

In addition to DAFs, AFD.LD and BAYESCAN SNPs showed a higher LD across Eurasian populations [$\bar{r_{\text{eurasia}}^{2}}$ (20 kb)] than climate‐associated SNPs (Figure 2b). Compared to the genome‐wide average LD (~0.12) and the 95th percentile (~0.34), the average LDs across the four sets of SNPs were BAYESCAN (~0.27); AFD.LD (~0.30); LFMM (~0.13); and GEMMA (~0.13).

Using the bins in which each set of SNPs showed the highest difference when compared to the genome average (Figure 2a), we found that low‐frequency GEMMA and LFMM variants (green and purple points) were mostly found in North Sweden, Russia, and other parts of central Asia (Figure 2c). On the other hand, AFD.LD (blue) and BAYESCAN (black) SNPs with DAFs between 0.2–0.3 and 0.3–0.4 showed the highest frequency in North Sweden (Figure 2c), while SNPs with DAFs between 0.7–0.8 and 0.6–0.7 showed a depletion in North Sweden (Figure 2c) and higher frequencies in central Europe (Appendix S4). In addition to differences in location, significant LFMM and GEMMA alleles showed much higher differences in mean Min.Tmp.Cld.M (~10°C) than AFD.LD and BAYESCAN alleles (~3.6°C) (Figure 2d). This is not surprising since GEA methods identify SNPs that explain more climate variation than the genome average.

### 3.3 High AFD and LD SNPs show a strong association with fitness QTL peaks and an enrichment at cis‐regulatory and nonsynonymous sites showing significant selective constraint

As mentioned earlier, alleles underlying local adaptation are expected to be enriched along sites that show significant evidence of function, but most importantly along QTL explaining fitness variation between populations (which provide direct evidence of local adaptation). To test the above, we looked at the distribution of SNPs across nonsynonymous/cis‐regulatory sites showing significant selective/functional constraint among Brassicaceae plants (Haudry et al., 2013) (phastCons > 0.8) and QTL explaining fitness variation between locally adapted Italy and Sweden populations (Ågren et al., 2013; Ågren & Schemske, 2012).

**Figure 3:**
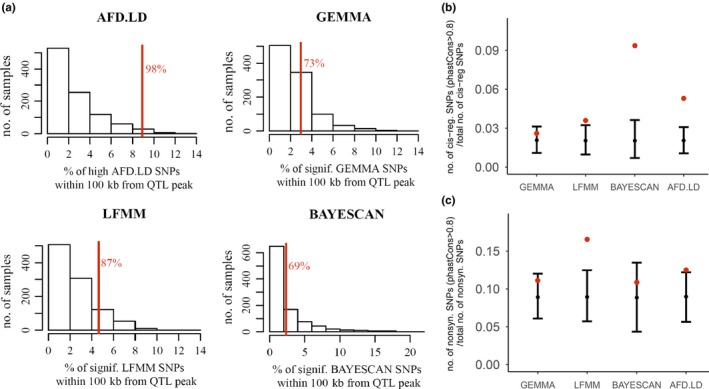
Testing for enrichment of candidate SNPs along QTL peaks explaining fitness variation between Italy and Sweden populations (Ågren et al., 2013) and cis‐regulatory/nonsynonymous sites showing significant selective constraint across nine Brassicaceae species (Haudry et al., 2013). (a) Comparing the observed proportion of candidate SNPs (red lines) within 100 kb of fitness QTL peaks, to a distribution of expected proportions derived using circular permutations. Significance was set at the 95th percentile. (b) The observed proportion of candidate SNPs (red dots) along cis‐regulatory sites showing significant selective constraint in relation to the expected proportion (in black are 95% intervals estimated using circular permutations). (c) The observed proportion of SNPs along nonsynonymous sites showing significant selective constraint

To examine direct evidence of local adaptation underlying SNPs identified by the four approaches, we first examined the proportion of SNPs at different distances from 20 QTL peaks (i.e., markers showing highest LOD score). Appendix S4 shows how the proportion of SNPs changes with distance from QTL peaks. Under the assumption that the proportion of SNPs showing significant evidence of local adaptation should be highest near LOD peaks, and decrease with distance, only LFMM and AFD.LD SNPs showed a negative association with distance (Appendix S4). The association of LFMM SNPs, however, was weaker (*R* ^2^ = .04, *p*‐value = .56) in comparison with AFD.LD SNPs (*R* ^2^ = .2, *p*‐value = .2), in addition to the proportion of AFD.LD SNPs being twice as high than LFMM SNPs at a close distance from QTL peaks (0–100 kb) (Appendix S4). The nonsignificant negative association observed across AFD.LD SNPs could be caused by the small number of points (*n* = 10) (Appendix S4). In addition to the strongest association, the proportion of AFD.LD SNPs near QTL peaks was significantly (>95% percentile) higher than expected by chance (Figure 3c). The second largest proportion relative to expectation was observed across LFMM SNPs (Figure 3c). LFMM SNPs within 100 kb of fitness QTL peaks showed a much lower mean AFD (~0.36) and LD (~0.10) than AFD.LD SNPs, which by definition are high in AFD and LD (AFD > 0.70 & LD > 0.19). This is not surprising given the low mean AFD and LD across all significant LFMM SNPs (Figure 1c,d).

SNPs that were significant according to BAYESCAN, showed a high AFD and LD (Figure 1c,d), but nonetheless they did not show a strong association (Appendix S4) and significant enrichment along fitness QTL, such as, high AFD.LD SNPs (Figure 3c). This may occur because significant BAYESCAN SNPs cannot capture all selection events underlying fitness QTL.

To examine where AFD.LD and BAYESCAN differed along genetic trade‐off QTL that were built using 20 fitness QTL (Ågren et al., 2013), we scanned for regions showing a high proportion of SNPs that were common between the two sets (AFD.LD and BAYESCAN) and SNPs that were unique to the AFD.LD set. As shown in Appendix S5, genetic trade‐off QTL that included regions that show high composite likelihood ratios for recent sweeps we see a high proportion of SNPs that are common to both approaches; on the other hand, along genetic trade‐off QTL where evidence of recent sweeps is almost absent (Appenices S6 and S7) we only observe SNPs that were unique to the AFD.LD set.

Finally, we examined the distribution of candidate SNPs underlying local adaptation across cis‐regulatory/nonsynonymous sites showing significant selective constraint. As shown in Figure 3b,c, the observed proportions of GEMMA SNPs were within the random expectation, while the proportions of LFMM and AFD.LD SNPs were higher than the expectation at both conserved cis‐regulatory and nonsynonymous sites. Finally, BAYESCAN SNPs were enriched only along conserved nonsynonymous sites (Figure 3a). For methods that showed an enrichment at conserved sites (cis‐regulatory and/or nonsynonymous sites), average LD in Eurasia ($\bar{r_{\text{eurasia}}^{2}}$) for BAYESCAN, LFMM, and AFD.LD SNPs was 0.27, 0.12, and 0.29, respectively. The average LD across LFMM SNPs was approximately the same as the genome‐wide mean at conserved cis‐regulatory and nonsynonymous sites (~0.12), while for BAYESCAN/AFD.LD SNPs was twice as high. Similarly, to the whole set of SNPs (Figure 2a), LFMM SNPs also showed a low mean DAF_eurasia_ (~0.13).

### 3.4 Genes underlying fitness QTL and controlling flowering time show significant evidence of local adaptation

Given the significant evidence of local adaptation and function underlying AFD.LD SNPs, we used them to detect potential genes that may underlie fitness QTL (note: We only focused on variants within 100 kb of their peaks), in addition to examining evidence of local adaptation underlying a list of ~170 genes affecting flowering time (Table S6). To further narrow down on SNPs that are more likely to underlie the fitness QTL examined, we only considered cis‐regulatory/nonsynonymous variation at conserved sites that segregated between the parents used to derive the RILs (Ågren et al., 2013). SNPs between the parental genomes were called in a previous study (Price et al., 2018).

**Figure 4:**
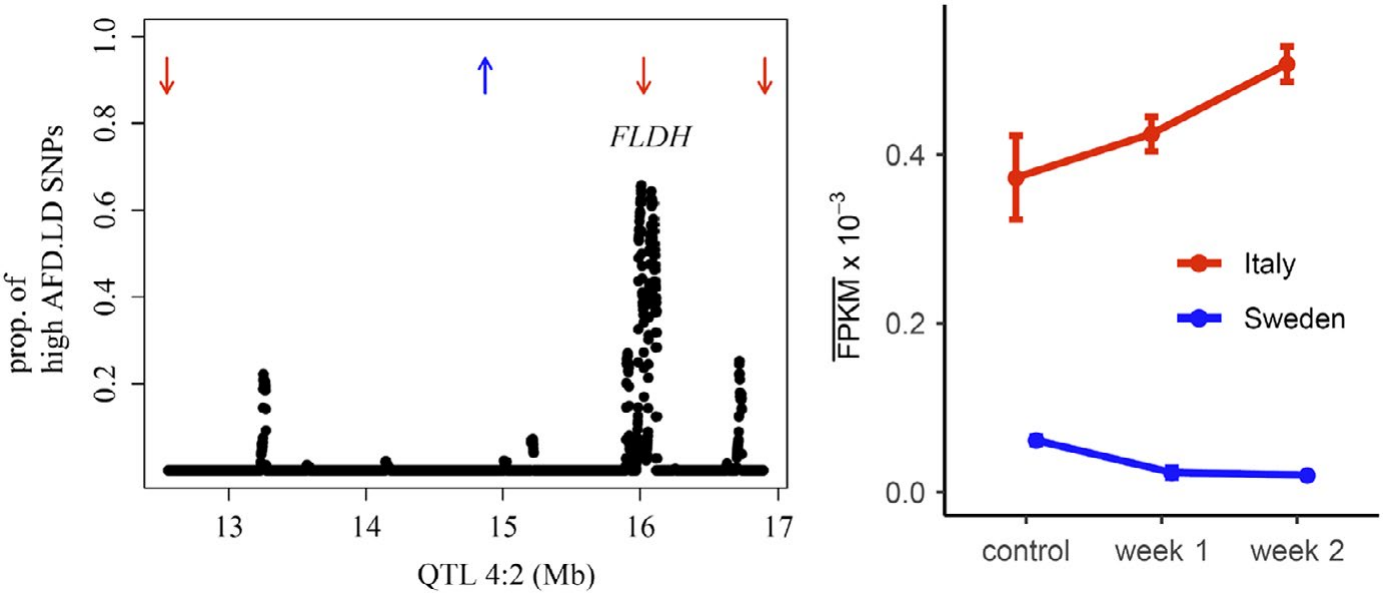
Significant evidence of local adaptation underlying the *FLDH* gene. *FLDH* is a negative regulator of ABA (Bhandari et al., 2010), found within genetic trade‐off QTL 4:2 (Ågren et al., 2013) and 100 kb from a fitness QTL peak (red and blue arrows represent QTL where the Sweden genotype had lower fitness in Italy and higher fitness in Sweden, respectively). The region including *FLDH* showed a high proportion of high AFD and LD (AFD.LD) SNPs, and furthermore, in Italy and Sweden plants *FLDH* showed strong expression GxE interactions under control (22°C) and cold acclimation conditions (4°C) for two weeks (FPKM: fragments per kilobase million)

Our analysis resulted in 24 genes within 100 kb of fitness QTL peaks and spanning three genetic trade‐off QTL (2:2, 4:2, and 5:5) (Appendix S8). Many of these were involved in interesting biological processes such as response to different abiotic stress factors and the abscisic‐acid signaling pathway which is important in abiotic stress response (Tuteja, 2007) (Appendix S8). Among these genes, two of them (AT4G33360 (*FLDH*) and AT4G33470 (*HDA14*)) showed strong expression G×E interactions [G×E interactions were identified in a previous study (Price et al., 2018)] when Italy and Sweden plants were grown under cold acclimation conditions (4°C) for two weeks (Gehan et al., 2015). Interestingly, *FLDH* is a negative regulator of the abscisic‐acid signaling pathway (Bhandari, Fitzpatrick, & Crowell, 2010). As shown in Figure 4, this gene was within a region of a genetic trade‐off QTL that showed a high proportion of AFD.LD SNPs. Furthermore, expression of *FLDH* under control and cold acclimation conditions was significantly lower in Sweden than Italy plants (Figure 4).

**Figure 5:**
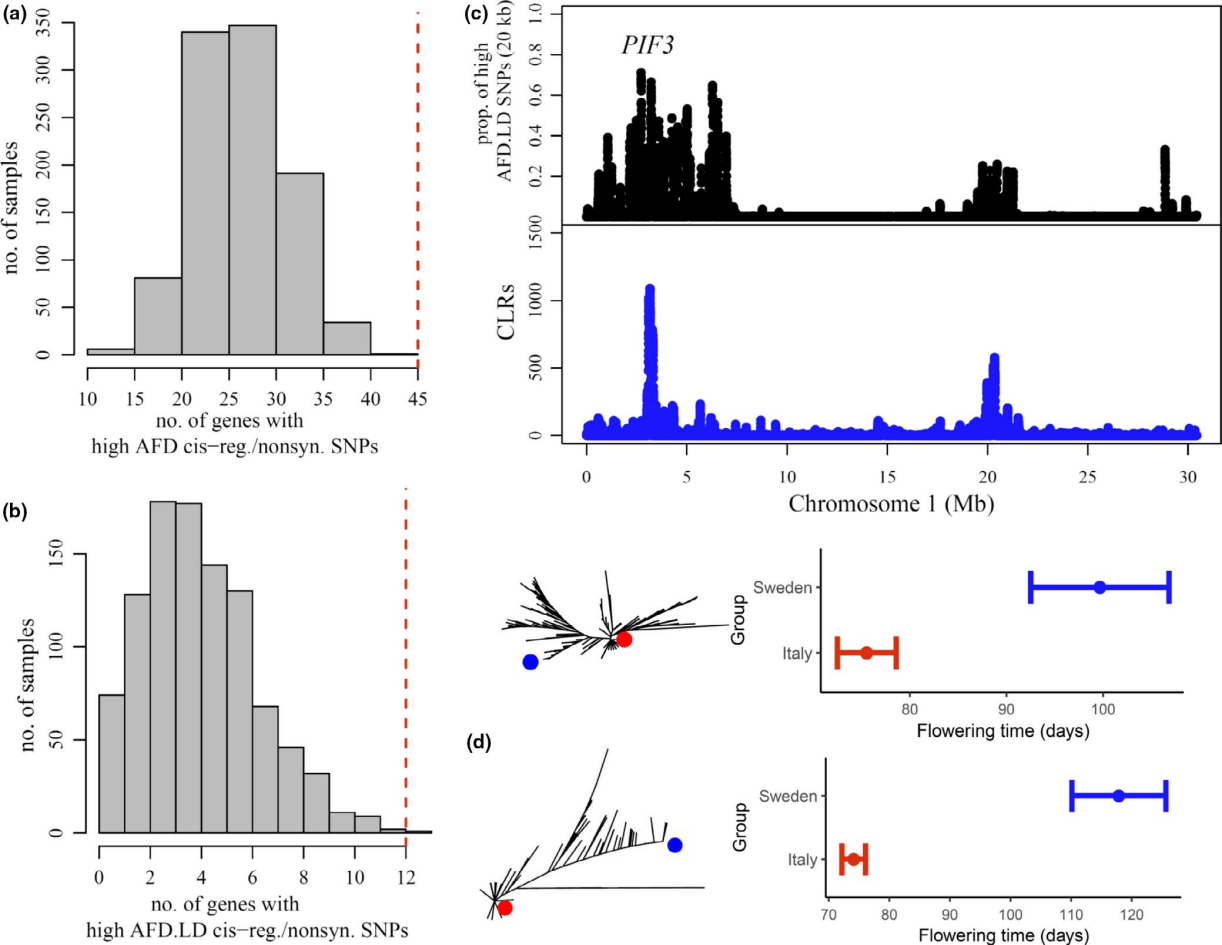
Genes known to affect flowering time show significant evidence of local adaptation along putative functional sites. (a) The number of flowering time genes containing cis‐regulatory/nonsynonymous SNPs showing a high AFD was significantly higher (>95th percentile) than expected by chance. Distribution of random numbers was derived using circular permutations. (b) The number of flowering time genes with a high AFD and LD cis‐regulatory/nonsynonymous SNPs was also significantly higher than expected by chance. (c) *PIF3* is a phytochrome interacting factor that has been found to affect flowering time (Oda et al., 2004) that was found underlying a region along chromosome 1 that showed the largest composite likelihood ratios (CLRs) for recent sweeps in Sweden, and windows with a high proportion of AFD.LD SNPs. A rooted phylogeny of the PIF3 coding region indicated that Eurasian accessions sharing the same allele as the Sweden parent (blue dot) show significantly higher flowering time than accessions sharing the same allele as the Italy parent (red dot). (d) *COL5* is another gene that has been found to affect flowering time (Hassidim, Harir, Yakir, Kron, & Green, 2009) and in which Eurasian accessions show significant genetic differentiation and segregation in flowering time. This gene is also found within previously identified flowering time QTL (FlrT‐5:4) (Ågren et al., 2017) in which the Sweden genotype was associated with longer flowering time in both Italy and Sweden

To examine whether genes affecting flowering time (Table S6) show significant evidence of local adaptation, we tested whether the number of such genes containing cis‐regulatory and/or nonsynonymous SNPs with a high AFD (Figure 5a), and a high AFD and LD (AFD.LD) (Figure 5b) was significantly higher than expected by chance (>95%). As shown in Figure 5a,b, the observed number of genes is significantly higher than the expectation. Among the 12 genes with high AFD and LD SNPs, we identified three [AT1G09530 (*PIF3*), AT2G21070 (*FIO1*), and AT5G57660 (*COL5*)] that contained such SNPs along conserved nonsynonymous sites. Among the three genes, *PIF3* was found along a chromosomal region that showed the highest CLR for a recent sweep in Sweden and a high density of AFD.LD SNPs (Figure 5c). Eurasian accessions sharing a similar allele as the Sweden parent showed longer flowering time than accessions sharing the same allele as the Italy parent (Figure 5c). The same pattern was observed when examining *COL5* (Figure 5d), a flowering time gene which was also found within a flowering time QTL (FlrT‐5:4, Table S5). According to FlrT‐5:4, the Sweden genotype was associated with longer flowering time in both Italy and Sweden (Ågren et al., 2017). In conjunction, with its overlap to a genetic trade‐off QTL (Ågren et al., 2017), it indicates a possible role in fitness trade‐offs. Studies have attributed flowering time variation within FlrT‐5:4 to *VIN3* (Ågren et al., 2017; 1001 Genomes Consortium, 2016). Although it may be an additional candidate, we did not find any significant genetic differentiation and selection along coding and cis‐regulatory sites of *VIN3*.

When examining flowering time genes with high *AFD* and LD SNPs along cis‐regulatory/nonsynonymous sites that did not show significant selective constraint, we identified an additional nine genes, four of which were found within flowering time QTL (FlrT): AT1G14920 (*GAI*); AT1G53090 (*SPA4*); AT1G79460 (*GA2*); AT2G22540 (*SVP*); AT2G28550 (*RAP2.7*); AT2G47700 (RFI2); AT4G32980 (*ATH1*‐FlrT4:1); AT5G24470 (*PRR5*‐FlrT5:2); and AT5G65060 (*MAF3*‐FlrT5:5). ATH1 was found in genetic trade‐off QTL 4:2, while gene MAF3 was found within genetic trade‐off QTL 5:5 and within 100 kb of fitness QTL peaks.

## 4 DISCUSSION

In the quest to study the genetic basis of local adaptation using genome‐wide associations with environment, linear mixed models have emerged as a powerful tool given their ability to account for population structure while testing for significant associations (Caye et al., 2019; Kang et al., 2010, 2008; Yu et al., 2006; Zhou & Stephens, 2012). Although they provide a robust statistical framework, the current study shows that such approaches may significantly limit our ability to understand the polygenic basis of local adaptation.

Both GEA methods (GEMMA and LFMM) resulted in SNPs that showed poor associations with QTL explaining fitness variation, in addition to low genetic differentiation and evidence of recent selection across locally adapted populations. The poor performance of GEA methods when examining populations isolated by distance has also been shown when using simulations (Lotterhos & Whitlock, 2015). In fact, *F*_ST_ ‐based approaches outperformed GEA methods in such instances (Lotterhos & Whitlock, 2015). In the current study, we show that SNPs exhibiting a significantly high *F_ST_* according to BAYESCAN, capture higher population genomic evidence of recent selection but fail to show a strong association with fitness QTL and a significant enrichment along regions with a high LOD score. Using a more lenient FDR cutoff may capture some of the missing SNPs across fitness QTL that do not contain strong CLRs for recent sweeps (Appenices S6 and S7).

SNPs that show extreme AFD and LD are likely to contain many false positives, especially if no other information is considered. These genomic signatures, however, show a promising future in identifying recent local adaptation within a statistical framework (Kemppainen et al., 2015). Such an approach can avoid several complications of GEA methods, including (a) errors in measures of environmental variables; (b) an increase in false positives if more than one environmental variable is needed to capture the genetic basis of local adaptation; and (c) difficulties in choosing the correct minimum allele frequency to avoid spurious associations.

*Arabidopsis thaliana,* however, is a simple, highly inbred species, and the populations we examined are separated by a very large geographic distance. GEA methods have been suggested to perform best across populations that do not exhibit a hierarchical population structure and isolated by large distances (De Villemereuil et al., 2014; Lotterhos & Whitlock, 2015). An example of such populations is ones found in Northern and Southern Sweden, which follow a two‐population island model (Huber et al., 2014). Examining the relation between SNPs showing significant associations with climate and other population genomic or field‐based evidence of local adaptation can shed further light on the ability of GEA methods under such scenarios. Furthermore, such analyses can be expanded to include species with different life‐history traits, such as the perennial European aspen, *Populus tremula* (Ingvarsson & Bernhardsson, 2019; Wang et al., 2018).

Despite the poor evidence of local adaptation and selection, SNPs identified by the GEA method LFMM showed an enrichment along sites showing significant selective constraint. Given the significant enrichment of such SNPs among low‐frequency‐derived alleles that show poor direct and indirect evidence of local adaptation, we raise caution when interpreting such results. Enrichment of climate‐associated SNPs along nonsynonymous sites (Hancock, Brachi, et al., 2011; Hancock, Witonsky, et al., 2011; Lasky et al., 2012) or the parallel occurrence of low‐frequency loss‐of‐function mutations showing significant associations with climate (Monroe et al., 2016, 2018) has been interpreted as evidence of adaptation. Although such signals may represent instances of adaptation, they can also be explained by neutral evolution, in which relaxed selection across specific climates results in the enrichment of independent loss‐of‐function mutations or nonsynonymous variation (Flowers, Hanzawa, Hall, Moore, & Purugganan, 2009; Zhen, Dhakal, & Ungerer, 2011; Zhen & Ungerer, 2008). In the context of local adaptation, these may represent instances of conditional neutrality, where in one environment expressing the gene has no significant impact on fitness. Some ways that could provide further support as to whether a recent loss‐of‐function mutation is adaptive are to compare LD or extended haplotype homozygosity (Sabeti et al., 2002) between individuals that have a loss‐of‐function mutation and ones that do not.

Among the genomic signatures of local adaptation examined, SNPs showing a high absolute allele frequency differentiation (AFD) and linkage disequilibrium (LD) between Italy and Sweden populations showed the strongest evidence of local adaptation and were enriched among nonsynonymous/cis‐regulatory variation at sites showing significant selective constraint. Using these SNPs, we identified a list of candidate genes underlying fitness QTL. One of these was *FLDH*, a negative regulator of abscisic‐acid signaling (Bhandari et al., 2010), that showed strong GxE interactions between Italy and Sweden plants under cold acclimation conditions. Abscisic‐acid signaling is known to play an important role in abiotic stress response (Tuteja, 2007), with many studies supporting a key role in local adaptation to climate (Kalladan et al., 2017; Keller, Levsen, Olson, & Tiffin, 2012; Lasky et al., 2014; Ristova, Giovannetti, Metesch, & Busch, 2018). In addition to abscisic‐acid signaling, our study provides further support for the important role of flowering time in local adaptation to climate. Among a list of genes that were experimentally shown to affect flowering time, we identified three genes (*PIF3*, *FIO1*, and *COL5*) that showed significant evidence of local adaptation and selective constraint along nonsynonymous sites. *FIO1* was previously shown to contain SNPs that showed significant associations with flowering time among natural Swedish lines (Sasaki, Zhang, Atwell, Meng, & Nordborg, 2015) and *COL5* was located within a QTL that explain flowering time variation among Sweden and Italy recombinant inbred lines (Ågren et al., 2017). Finally, *PIF3,* a transcription factor that interacts with phytochromes (Soy et al., 2012), has been implicated in multiple biological processes including early hypocotyl growth (Monte et al., 2004), photomorphogenesis (Dong et al., 2017), flowering time (Oda, Fujiwara, Kamada, Coupland, & Mizoguchi, 2004), and regulation of physiological responses to temperature (Jiang et al., 2017).

Interestingly, *PIF3* was found within a large region that showed significant evidence of local adaptation. Regions of high divergence may involve a single causative variant, or a group of linked genes that interact with *PIF3* and were under selection because they contributed to building an advantageous phenotype (Barton & Bengtsson, 1986; Yeaman & Whitlock, 2011). Although such “Islands of high divergence” can be the result of local adaptation, they can also be formed through nonadaptive processes (Pennisi, 2014), and therefore, caution should be exercised when drawing any conclusions.

When ignoring selective constraint, we identify a list of addition flowering time genes showing significant evidence of local adaptation along nonsynonymous/cis‐regulatory sites. Genes such as SVP and MAF3 were previously associated with flowering time variation among natural *Arabidopsis* accessions (Caicedo, Richards, Ehrenreich, & Purugganan, 2009; Sasaki et al., 2015), and MAF3 showed strong allelic variation along a multivariate climate gradient (Lasky et al., 2012). Although adaptation may involve sites that are not deeply rooted and/or under strong selective constraint, including additional plant genomes when estimating sequence conservation across species may increase our power to detect selectively important regions. As shown by studies examining adaptation in species ranging from bacteria (Maddamsetti et al., 2017) to birds (Sackton et al., 2019), addressing selective constraint can improve our understanding of its genetic basis.

To sum up, the current study identifies candidate genes and life‐history traits that may underlie adaptation of Arabidopsis populations to local environments. Furthermore, it shows that understanding organismal adaptation to local environments is a very complex venture, where several lines of evidence are needed to obtain a comprehensive and well‐supported picture.

## CONFLICT OF INTEREST

The authors declare that they have no conflict of interest.

## AUTHOR CONTRIBUTIONS

Nicholas Price designed research, performed research, analyzed data, and wrote the paper. Lua Lopez and Adrian E. Platts performed research and reviewed the paper. Jesse R. Lasky reviewed the paper.

## Data Availability

In our dryad submission, we included:
The false discovery rate (FDR) associated with SNPs used to identify significant associations with Minimum Temperature of Coldest Month using GEMMA and LFMM The FDR associated with SNPs used to identify instances of significant allele frequency differentiation (approximated by *F*_ST_) between Italy and Sweden populations using BAYESCAN. Absolute nonreference allele frequency differentiation (AFD) estimated between Italy and Sweden populations Derived allele frequency divergence (DAF) across 875 Eurasian accessions Linkage disequilibrium estimated using 65 Italy and Sweden accessions and 875 Eurasian accessions Composite likelihood ratios (CLRs) for recent sweeps in North Sweden populations Climate data for all the 1,135 *A. thaliana* re‐sequenced genomes that have been recently published (1001 Genomes Consortium, 2016). Conserved coding regions Conserved noncoding regions Private link: https://datadryad.org/stash/share/962vtPUZ9tTuwtrRKA1px_WegXVYYEYsvxAWa_6If_0
