# Supplementary material for: In the presence of population structure: From genomics to candidate genes underlying local adaptation
Source: Ecol Evol. 2020 Feb 12;10(4):1889–904. doi: 10.1002/ece3.6002 (PMC7042746; doi:10.1002/ece3.6002)
Supplement: Supplementary file 2 [file ECE3-10-1889-s002.docx]

Appendix 1. The resulting p-value distribution underlying associations to Minimum Temperature of Coldest Month (Min.Tmp.Cld.M) after accounting for population structure using GEMMA (Zhou and Stephens 2012).

Appendix 2. Applying LFMM 2 (Caye, et al. 2019) to identify allele associations to Min.Tmp.Cld.M. On the left hand side, the figure explains the amount of genetic variance explained by a series of 20 principal components. Using eight principal components as latent factors (K=8) in LFMM 2 resulted in a p-value distribution (right) that was enriched in low p-values and was uniform at higher p-values. Furthermore, using K=8 resulted in a similar number of significant SNPs as GEMMA.

0.7-0.8

0.2-0.3

0.3-0.4

0.6-0.7

Appendix 3. The location of derived AFD.LD (blue) and BAYESCAN (black) derived alleles at frequencies that showed the highest difference when compared to the genome average (Figure 2A). High AFD. LD SNPs (blue) at derive allele frequencies (DAFs) between 0.2-0.3 were most frequent across North Sweden populations, while alleles at DAFs between 0.7-0.8 showed a depletion in North Sweden and higher frequency across central Europe. The same approximate pattern was observed across significant BAYESCAN SNPs with intermediate DAFs (0.3-0.4) and high DAFs (0.6-0.7).

Appendix 4. The relation between SNPs showing significant evidence of local adaptation and distance from 20 fitness QTL peaks (Ågren, et al. 2013). The y-axis represents the proportion of SNPs within non-overlapping 100 kb windows, that started from the marker location with the highest LOD score and moving outwards to 1000 kb. The windows were non overlapping and spanned the region left and right of the QTL peak; therefore, in total these windows spanned 200 kb. AFD.LD SNPs were the only ones that showed the highest proportion closest to the QTL peak and strongest negative correlation with distance. Although not significant, this maybe cause by the small number of samples.

Appendix 5. Evidence of recent selection and allele frequency differentiation along a genetic tradeoff QTL that was previously identified by Ågren, et al. (2013) (Ågren, et al. 2013). The top panel includes 1kb windows of Composite likelihood Ratios (CLR) for recent sweeps in North Sweden. The CLR expected under a simple model of neutral evolution was previously estimated to be ~100 (Price et al., 2018). The arrows are the locations of QTL peaks (highest LOD scores) in which the Swedish genotype showed significantly lower (red) or higher (blue) fitness than the Italy genotype. The middle panel depicts the proportion of SNPs within a 20kb window, that showed a significantly high F_ST_ according to BAYESCAN (Foll and Gaggiotti 2008) and a high absolute frequency divergence and linkage disequilibrium (AFD.LD) between Italy and Sweden populations. The lower panel depicts the proportion of SNPs within a 20kb window that showed a high absolute frequency divergence and linkage disequilibrium (AFD.LD) but were not identified by BAYESCAN.

Appendix 6. The number 2 along the top panel indicates that the same QTL was identified twice during the three-year experiment (Ågren, et al. 2013).

Appendix 7.

Appendix 8. A list of 24 genes with cis-regulatory and/or nonsynonymous SNPs found in conserved regions and within 100 kb of fitness QTL peaks. The genes identified were close to fitness QTL that were part of three genetic tradeoff QTL (GT QTL), 2:2, 4:2, 5:5 (Ågren, et al. 2013). The biological processes these genes were retrieved from the TAIR database.

| Gene | GT QTL | Biological process |
| --- | --- | --- |
| AT2G36230 | 2:2 | histidine biosynthetic process, tryptophan biosynthetic process |
| AT2G44210 | 2:2 | Unknown |
| AT4G33140 | 4:2 | deoxyribonucleotide catabolic process |
| AT4G33150 | 4:2 | This is a splice variant of the LKR/SDH locus. It encodes a bifunctional polypeptide lysine-ketoglutarate reductase and saccharopine dehydrogenase involved in lysine degradation. There is another splice variant that encodes a mono saccharopine dehydrogenase protein. Gene expression is induced by abscisic acid, jasmonate, and under sucrose starvation. |
| AT4G33200 | 4:2 | actin filament organization, actin filament-based movement, nuclear migration,regulation of establishment or maintenance of cell polarity regulating cell shape |
| AT4G33240 | 4:2 | endomembrane system organization, phosphatidylinositol phosphorylation, pollen development, vacuole organization |
| AT4G33350 | 4:2 | protein folding, protein transport |
| AT4G33360 | 4:2 | farnesol metabolic process, negative regulation of abscisic acid-activated signaling pathway, terpenoid metabolic process |
| AT4G33380 | 4:2 | Uknown |
| AT4G33410 | 4:2 | membrane protein proteolysis, signal peptide processing |
| AT4G33420 | 4:2 | hydrogen peroxide catabolic process, oxidation-reduction process, response to oxidative stress |
| AT4G33470 | 4:2 | histone deacetylation, tubulin deacetylation |
| AT5G64730 | 5:5 | Unknown |
| AT5G64860 | 5:5 | unknown (DPE2 cold acclimation) |
| AT5G64930 | 5:5 | defense response, jasmonic acid and ethylene-dependent systemic resistance, jasmonic acid mediated signaling pathway, leaf senescence, photoperiodism, flowering, plant-type hypersensitive response, response to other organism, sugar mediated signaling pathway, systemic acquired resistance, trichome morphogenesis |
| AT5G65020 | 5:5 | phloem sucrose unloading, polysaccharide transport, primary root development,response to cold, response to heat, response to salt stress, response to water deprivation |
| AT5G65274 | 5:5 | Uknown |
| AT5G65450 | 5:5 | protein deubiquitination, ubiquitin-dependent protein catabolic process |
| AT5G65460 | 5:5 | abscisic acid-activated signaling pathway, hyperosmotic salinity response,negative regulation of transcription, DNA-templated, response to chitin, response to cold |
| AT5G65530 | 5:5 | defense response to fungus, protein autophosphorylation, protein phosphorylation,trichome branching |
| AT5G65683 | 5:5 | gravitropism, root development |
| AT5G65690 | 5:5 | Gluconeogenesis |
| AT5G66890 | 5:5 | defense response |
| AT5G66960 | 5:5 | Proteolysis |

References

Ågren J, Oakley CG, McKay JK, Lovell JT, Schemske DW. 2013. Genetic mapping of adaptation reveals fitness tradeoffs in Arabidopsis thaliana. Proc Natl Acad Sci U S A 110:21077-21082.

Caye K, Jumentier B, Lepeule J, Francois O. 2019. LFMM 2: Fast and Accurate Inference of Gene-Environment Associations in Genome-Wide Studies. Mol Biol Evol 36:852-860.

Foll M, Gaggiotti O. 2008. A genome-scan method to identify selected loci appropriate for both dominant and codominant markers: a Bayesian perspective. Genetics 180:977-993.

Zhou X, Stephens M. 2012. Genome-wide efficient mixed-model analysis for association studies. Nat Genet 44:821-824.
